# T161. HEARING VOICES AMONG INDIGENOUS MAASAI WOMEN IN TANZANIA: IMPLICATIONS FOR GLOBAL MENTAL HEALTH

**DOI:** 10.1093/schbul/sby016.437

**Published:** 2018-04-01

**Authors:** Neely Myers, Luca Pauselli, Michael Compton

**Affiliations:** 1 Southern Methodist University; 2 College of Physicians and Surgeons, Columbia University/New York State Psychiatric Institute; 3 College of Physicians and Surgeons, Columbia University

## Abstract

**Background:**

Studies of the health of indigenous and tribal people can shed light on health inequalities with implications for global mental health. Almost no previous studies of mental health among the Maasai in Kenya or Tanzania or even pastoralist groups in general are available, with the exception of one mentioning the stress of rural-to-urban migration. While engaged in ethnographic research in 2013, Myers interviewed 13 Maasai women in northern Tanzania about their everyday lives. Interested in the phenomenology of voice-hearing, she also asked them if they heard voices. Eleven of the women (85%) reported regularly hearing voices that they found to be distressing. Myers returned in 2015 to collect data from a larger sample, which has resulted in this report.

**Methods:**

For this study, we used a convenience sample (n=73) of females taken from a broader study whose eligibility criteria included being a Maasai person living in the Arusha Region of Tanzania and over the age of 18. We excluded people who reported being psychiatric patients or family members of patients being seen by the local mental health coordinator to create a nonclinical community sample. This project conducted an initial survey to: 1) estimate the community prevalence of voice-hearing, or auditory verbal hallucinations (AVHs) in this specific population; and, 2) examine any demographic correlates and two specific hypothesized correlates based on previous literature about voice-hearing (e.g., psychological stress and potentially traumatic events).

**Results:**

The prevalence of AVHs in this community sample was quite high compared to other studies in sub-Saharan Africa, at 34.3%. There were no differences between participants who did and did not experience AVHs in terms of demographics, but those experiencing AVHs had a statistically significantly higher level of psychological distress (30.1, SD=6.0, compared to 25.1, SD=6.0), with a Cohen’s d effect size of .87. Even though a numerical difference was observed in terms of potentially traumatic events (4.6, SD=2.1, compared to 3.8, SD=2.3), this difference was not statistically significant (d=.38).

**Discussion:**

Hearing distressing voices may be an indicator of mental ill health that is easily recognizable to community health workers and brings much-needed attention to communities in need. Maasai women face tremendous social adversity in this time of rapid social, economic, and climate change in the region. Evidence for a link between stressful life events, social disadvantage, and the development of psychotic symptoms is strong in Europe, but not as well-developed in this region. The high level of psychosocial stress and AVHs in our sample may also be indicative of extreme social adversity. Maasai women have been historically disenfranchised since the advent of colonial and postcolonial policies favoring men. Women in this region also experiences extreme states of deprivation, including a 21.5 year gap in life expectancy at birth compared to the local population (for all Maasai), 81% severe food insecurity, and a rate of 59% for the stunted growth of children (as compared to their neighbors, the Meru, with 21% of children with stunted growth). Due to local livelihood insecurity, the age of first marriage has been decreasing, resulting in increased pressure on women. This analysis contextualizes these findings and calls for further research on the epidemiology of voice-hearing in this region, as well as further work on the phenomenology of these AVHs so that we can best understand how to address them and improve mental health outcomes for this marginalized group.

